# Effects of Surgery Combined with Different Chemotherapy on Matrix Metalloproteinase-9 and Tissue Inhibitors of Metalloproteinase-1 in Children with Neuroblastoma

**DOI:** 10.1155/2022/8319221

**Published:** 2022-07-05

**Authors:** Zilong Qi, Xinning Wang, Liang Yin, Kun Ma, Lei Chen, Jinmin Li

**Affiliations:** Department of Pediatric Surgery, Cangzhou Central Hospital, Cangzhou, China

## Abstract

**Background:**

Neuroblastoma (NB) is a common extracranial malignancy in children and accounts for 15% of all cancer-related deaths in children, with the 5-year survival of patients in an advanced stage being lower than 40%. Preoperative adjuvant chemotherapy has been reported to facilitate surgical resection and improve the 2-year survival of patients.

**Objective:**

To analyze the efficacy of surgery plus different chemotherapy on children with NB and to investigate the correlation of matrix metalloproteinase-9 (MMP-9) and tissue inhibitors of metalloproteinase-1 (TIMP-1) with chemotherapy efficacy.

**Methods:**

From April 2005 to May 2017, a total of 92 cases of NB treated in our hospital were assessed for eligibility and recruited. They were assigned at a ratio of 1: 1 to receive either CAV (cyclophosphamide + vincristine + adriamycin) (group A) and EP (etoposide + cisplatin) alternately or TOPO (topotecan) + CTX (cytoxan) + CiE (etoposide + cisplatin) + CPV (cyclophosphamide + pirarubicin + vincristine) (group B). The outcome measures include chemotherapy efficacy, surgical resection rates, complications, 2-year recurrence, and 2-year survival. The levels of NK cells, CD4+/CD8+ cells, MMP-9, TIMP-1, and urine catecholamine (VMA) in peripheral blood of patients before and after initial chemotherapy were determined to analyze the correlation of MMP-9, TIMP-1, and VMA with the efficacy of chemotherapy.

**Results:**

The two groups had similar efficacy (84.00% vs. 95.24%) and surgical resection rates (60.00% vs. 61.90%) after the initial chemotherapy (*P* > 0.05). Surgery for all eligible patients was successful after second chemotherapy. All eligible patients showed myelosuppression after chemotherapy, including 48 cases with stages I-II (52.17%) and 44 cases with stages III-IV (47.83%). The ratio of CD4+/CD8+ cells, MMP-9, TIMP-1, and VMA expression levels in peripheral blood of patients decreased (*P* < 0.05) after chemotherapy, and the ratio of CD4+/CD8+ cells was further reduced after surgery (*P* < 0.05), while natural killer (NK) cells levels increased (*P* < 0.05). However, intergroup differences were absent in the incidence of myelosuppression, CD4+/CD8+ cell ratio, NK cells, MMP-9, TIMP-1, and VMA expression levels (*P* > 0.05). MMP-9 and TIMP-1 were positively correlated with VMA (*P* < 0.05), and the expression levels of MMP-9 and TIMP-1 and VMA after chemotherapy were negatively correlated with chemotherapy efficiency (*P* < 0.05). Patients with high expressions of MMP-9, TIMP-1, and VMA were associated with lower 2-year survival versus those with low expressions (*P* < 0.05).

**Conclusion:**

Surgery plus chemotherapy for children with NB yields a promising clinical efficacy and a favorable surgical resection outcome. MMP-9 and TIMP-1 may be the potential biological indicators for chemotherapy efficiency and have a reference value for following surgical treatment of patients.

## 1. Introduction

Neuroblastoma (NB), also known as neurocytoma, is one of the most common extracranial malignant solid tumors in children, accounting for 6%–10% of all pediatric tumors [1, 2]. It accounts for 15% of all childhood cancer-related deaths and is the embryonal tumor with the lowest 5-year survival [3]. Although treatment approaches such as chemotherapy, surgery, and stem cell transplantation achieved improved prognosis for patients, patients in the advanced stage are associated with a 5-year survival lower than 40% [4, 5].

Surgery is the mainstay for the treatment of NB, and the resection of the primary lesion is of great significance for improving the therapeutic efficiency and prognosis and prolonging survival [6]. However, NB is associated with a high degree of malignancy and a big tumor size; nearly 2/3 of the patients are predisposed to multiple early metastases, with unfavorable complete surgical resection outcomes [7]. At present, controversy exists in the timing and scope of surgery for high-risk patients, which necessitates the amelioration of the treatment modalities. Previous research studies have reported that preoperative chemotherapy can reduce the primary lesion to facilitate surgical resection. Zhang et al. [8] used preoperative OPAC chemotherapy to improve surgical resection of NB with stages III and IV to 89% and 79%, respectively. Johnston et al. [9] also reported better surgical resection (60%) of NB after preoperative chemotherapy. The combination of surgery and chemotherapy in children with NB has been marginally explored, and the evaluation of preoperative chemotherapy efficiency mostly relies on the iconography observation of tumor size, which is impractical for routine analysis. Matrix metalloproteinase-9 (MMP-9) and tissue inhibitors of metalloproteinase-1 (TIMP-1) play important roles in the proliferation and metastasis of NB tumor cells [10, 11].

Accordingly, this study was conducted to analyze the efficacy of surgery plus different chemotherapy on children with NB and to investigate the correlation of MMP-9 and TIMP-1 with chemotherapy efficacy.

## 2. Data and Methods

### 2.1. Research Subjects

From April 2005 to May 2017, a total of 92 NB patients aged 1–15 years admitted to our institution which includes 61 males and 31 females were assessed for eligibility and recruited. Inclusion criteria were as follows: all patients were diagnosed with NB in the International Neuroblastoma Staging System (INSS) stages III-IV by pathology or by bone marrow hemocytology with unremovable tumors at first diagnosis and all patients' conditions were evaluated by clinicians and treated with preoperative chemotherapy combined with surgical resection, with complete medical records and follow-up data. Exclusion criteria were as follows: patients with other severe diseases or tumors, with coagulation disorders, with autoimmune diseases or long-term use of immunosuppressants, and with communication disorders or cognition impairment. This study has been approved by the ethical committee of our hospital, and all guardians of the patients have signed the informed consent and cooperated with medical staff to complete relevant diagnosis and treatment.

### 2.2. Therapies

Of the 92 eligible patients included in this study, 50 patients receiving alternative CAV or EP regimens were in group A and 42 eligible patients receiving TOPO + CTX + CiE + CPV regimen were assigned to group B. The efficacy of chemotherapy was evaluated. In the case of effective chemotherapy results with resectable tumors after assessments, the patients were given surgery and postoperative chemotherapy was intensified. Otherwise, CV chemotherapy was continued for 2–4 courses, followed by one course of CE treatment with the dose of DDP adjusted to 6.6 mg/kg i.v. gtt d1–d3 and the dose of VP16 adjusted to 5 mg/kg i.v. gtt d1–d3 if the CV regimen was ineffective. The specific chemotherapy scheme is given in Table 1.

### 2.3. Analysis of Chemotherapy Efficacy

The efficacy of chemotherapy in the two groups was observed and recorded. The efficacy of chemotherapy was divided into complete response (CR), partial response (PR), stable disease (SD), and progressive disease (PD). CR refers to all found lesions that were disappeared and the condition lasts for more than 4 weeks. PR refers to the sum of the largest single diameter of the tumor that was reduced by more than 30% and the condition lasts for more than 4 weeks. SD refers to the sum of the largest single diameter of the tumor that did not decrease by more than 30% or the increase did not exceed 20%, and the condition lasts 4 weeks. PD refers to the sum of the largest single diameter of the tumor increased by more than 20% or new lesions appeared.

### 2.4. Outcome Measures

The range of surgery, complications of initial chemotherapy, 2-year recurrence, and 2-year survival of the patients were recorded. The levels of NK cells, CD4+/CD8+ cells, MMP-9, TIMP-1, and urine vanillylmandelic acid (VMA) in peripheral blood of patients before and after initial chemotherapy were determined to analyze the correlation of MMP-9, TIMP-1, and VMA with the efficacy of chemotherapy.

### 2.5. Detection Methods

Enzyme-linked immunosorbent assay (ELISA) was used to determine the expression levels of MMP-9 and TIMP-1 in peripheral blood of patients, and the specific steps were following the kit instruction. MMP-9 and TIMP-1 assay kits were purchased from Abcam in the United States, with article no. of Ab100610 and ab187394, respectively. Urine VMA levels in both groups were determined by a high-performance liquid chromatography-mass spectrometer (LC-MS, ThermoFisher). Backman CytoFLEX flow cytometry (Beckman Coulter) was used to determine the levels of NK cells and CD4+/CD8+ cells in peripheral blood. Urine samples were random urine in 24 hours, and peripheral blood samples were morning fasting blood.

### 2.6. Statistical Analysis

SPSS 19.0 (Chicago, IL, the United States) was applied for statistical analysis. The distribution of outcomes was evaluated and presented as mean (SD) for continuous outcomes and frequency (%) for categorical outcomes. Normality was evaluated by the Kolmogorov–Smirnov test. If data were not normally distributed, nonparametric corollary for continuous outcomes and the *χ*^2^ test for categorical outcomes were used. Group comparisons of baseline participant characteristics were made using a *t*-test or nonparametric corollary for continuous outcomes and a *χ*^2^ test for categorical outcomes. Spearman and Pearson test was used for correlation analysis. The K-M curve and log-rank test were used to analyze the survival of patients. GraphPad Prism 8.0 (La Jolla, CA) was used for image rendering. An analysis of covariance (ANCOVA) model adjusting for the baseline value of the outcome was performed with the treatment group and time (baseline/post-intervention) as factors. For within-group difference, the changes in MMP from baseline to week 9 were examined by a paired *t*-test, with a level of significance set at *P* < 0.05. Repeated measures of ANCOVA on the trial outcomes were a 2 × 2 (time: baseline, postintervention) analysis.

## 3. Results

### 3.1. General Data

In this study, 92 patients were included as per the inclusion and exclusion criteria, including 61 males and 31 females, aged (6.6 ± 1.9) years. Among them, there were 38 cases in the INSS stage III and 54 cases in stage IV. Other specific data are given in Table 2. There was no significant statistical difference in the general data of patients in group A and group B (*P* > 0.05).

### 3.2. Chemotherapy Efficacy

The total effective rate was 84.00% in group A and 95.24% in group B, and there was no significant difference between the two groups (*P* > 0.05), as given in Table 3.

### 3.3. Surgical Resection

The lesion resection status of the patients was obtained from the surgical records. The resection rate of the patients in group A after initial chemotherapy was 60.00% and that in group B was 61.90%. There were no significant difference between the two groups (*P* > 0.05). After the second chemotherapy, all patients successfully received surgery, as given in Table 4.

### 3.4. Analysis of Complications

The incidence of myelosuppression after chemotherapy was analyzed. All patients showed myelosuppression after chemotherapy, including 48 patients with stages I-II (52.17%) and 44 patients with stages III-IV (47.83%). The results of peripheral blood immune cell classification also showed that the CD4+/CD8+ cell ratio in peripheral blood of patients after chemotherapy decreased (*P* < 0.05), and the ratio of CD4+/CD8+ cells was further reduced after surgery (*P* < 0.05), while NK cells levels were increased (*P* < 0.05). However, there was no significant difference in the incidence of myelosuppression, CD4+/CD8+ cell ratio, and NK cell level between the two groups (*P* > 0.05) (Table 5).

### 3.5. Changes of MMP-9, TIMP-1, and VMA Levels before and after Chemotherapy

**Figure 1:**
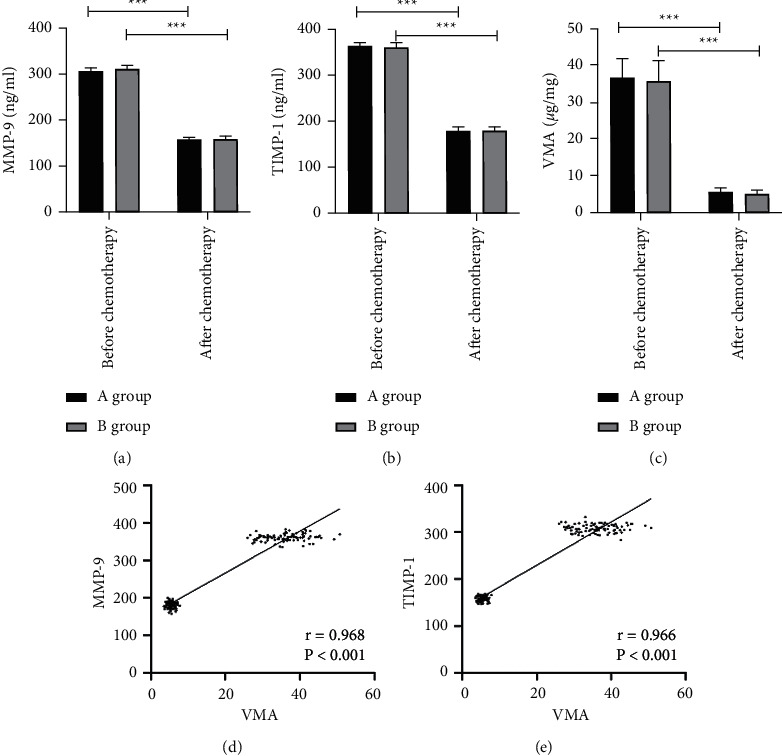
Changes of MMP-9 and TIMP-1 levels before and after chemotherapy. (a) MMP-9 level decreased significantly after chemotherapy. (b) TIMP-1 level decreased significantly after chemotherapy. (c) VMA level decreased significantly after chemotherapy. (d) Correlation analysis of VMA and MMP-9. (e) Correlation analysis of VMA and TIMP-1. ^*∗∗∗*^*P* < 0.001. MMP-9, matrix metalloproteinase-9; TIMP-1, tissue inhibitors of metalloproteinase-1; VMA, vanillylmandelic acid.

After chemotherapy, MMP-9, TIMP-1, and VMA levels in peripheral blood of patients in the two groups were significantly decreased (*P* < 0.05), but intergroup differences in MMP-9, TIMP-1 and VMA levels between group A and group B before and after chemotherapy were absent (*P* > 0.05). Pearson correlation analysis showed that both MMP-9 and TIMP-1 were positively correlated with VMA (*P* < 0.05) (Figure 1).

### 3.6. The Correlation of MMP-9, TIMP-1, and VMA with the Efficacy of Chemotherapy after Chemotherapy

**Figure 2:**
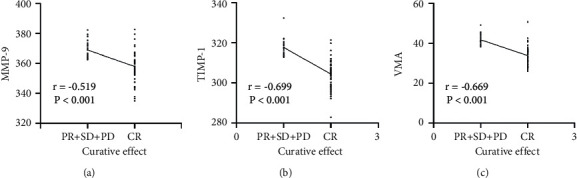
The correlation of MMP-9, TIMP-1, and VMA with the efficacy of chemotherapy after chemotherapy. (a) Correlation analysis of MMP-9 and chemotherapy efficacy after chemotherapy. (b) Correlation analysis of TIMP-1 and chemotherapy efficacy. (c) Correlation analysis of VMA and chemotherapy efficacy. MMP-9, matrix metalloproteinase-9; TIMP-1, tissue inhibitors of metalloproteinase-1; VMA, vanillylmandelic acid.

In this study, all patients who achieved CR in chemotherapy successfully underwent complete resection. Therefore, the patients were divided into a CR group and a PR + SD + PD group. Spearman correlation analysis showed that the expression levels of MMP-9, TIMP-1, and VMA after chemotherapy were negatively correlated with the efficacy of chemotherapy (*P* < 0.05) (Figure 2).

### 3.7. The Correlation of MMP-9, TIMP-1, and VMA with the Survival of Patients after Chemotherapy

**Figure 3:**
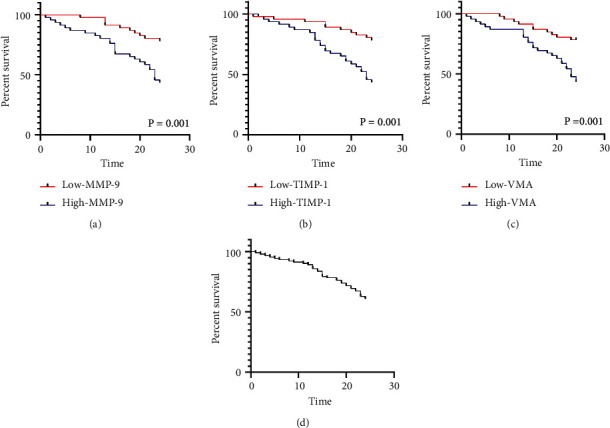
The correlation of MMP-9, TIMP-1, and VMA with the survival of patients after chemotherapy. (a) Correlation between MMP-9 and 2-year survival of patients. (b) Correlation between TIMP-1 and 2-year survival of patients. (c) Correlation between VMA and 2-year survival of patients. (d) 2-year survival curve. MMP-9, matrix metalloproteinase-9; TIMP-1, tissue inhibitors of metalloproteinase-1; VMA, vanillylmandelic acid.

Patients were divided into a high expression group and a low expression group according to the median values of MMP-9, TIMP-1, and VMA after chemotherapy. The K-M curve and log-rank test analysis showed that patients with high expressions of MMP-9, TIMP-1, and VMA were associated with lower 2-year survival versus those with low expressions (*P* < 0.05) (Figure 3).

## 4. Discussion

INSS is one of the most commonly used NB staging systems in clinical practice. Patients with NB in the INSS stages III-IV usually suffer from tumor infiltration or lymph node and distant metastases, and simple surgical treatment is considered insufficient for complete removal of the lesion, resulting in poor prognosis [12, 13]. Prior research has found that preoperative adjuvant chemotherapy can effectively facilitate surgical resection and prolong the 5-year survival of the patients [14]. However, the efficacy of the combination of surgery and chemotherapy for NB has been marginally explored, and there has been no unified view on preoperative chemotherapy for advanced NB patients. With the increasingly serious multidrug resistance of tumors and the continuous development of chemotherapy drugs, there exists an urgent need to analyze the differences in the efficacy of current preoperative chemotherapy in NB.

This study compared the efficacy of two preoperative chemotherapy regimens (CAV and EP alternative therapy or TOPO + CTX + CiE + CPV therapy) in NB. In the second scheme, the dosage of CTX, VCR, and VP16 was adjusted, and TOPO and pirarubicin were added to replace ADM. The latter three drugs all acted on DNA or RNA to exert antitumor effects [15–17]. The results of this study showed that there was no difference in the efficacy of chemotherapy and the incidence of myelosuppression between the two schemes. The regimens had significant inhibitory effects on the cellular immune function of patients, as evidenced by decreased CD4+/CD8+ cell ratio. In this study, the level of NK cells increased significantly after chemotherapy, and the cellular immune function of patients was further weakened by the further stressful stimulus of surgery. Similar results have been found in many studies, in which the cellular immune function of cancer patients is reduced after chemotherapy and surgical treatment [18, 19]. In the study of Xie and Wang [20], chemotherapy plus surgery mildly inhibits the cellular immune function of NB patients versus chemotherapy alone, and the recurrence of NB is closely related to the decreased immune function of patients. This also reflects the advantages of the combination approach for the treatment of NB.

In addition, the results of the present study showed no statistical differences in the surgical resection range between the two groups. In the study of Valteau-Couanet and Kushner et al. [21, 22], CAV and EP regimens are suitable for preoperative treatment of NB patients, and about 80% of patients can get a complete or partial response after receiving the chemotherapy combined with surgical treatment. However, the treatment of this regimen only lasts for 5 courses, as further treatment is associated with unimproved efficacy and increased toxicity. In this study, CAV and EP were alternated for 5 courses of treatment, while TOPO + CTX + CiE + CDV were alternated for 6 courses of treatment. This may suggest another option for patients with a strong constitution. Notwithstanding a longer chemotherapy cycle, TOPO + CTX + CiE + CDV adopts a combination of more drugs, which can effectively reduce the incidence of drug resistance [23].

Similar to the research results by Xie and Wang [20], the results of this study also showed decreased expression of MMP-9 and TIMP-1 after chemotherapy. MMPs usually form complexes with TIMPs in noncovalent bonds, which inhibits the tumor metastasis and the transformation of malignant phenotypes [24, 25]. Moreover, reduced levels of VMA in peripheral blood after chemotherapy were observed. VMA is one of the most commonly used indicators in the diagnosis of NB, which is related to high-risk characteristics of NB and can monitor chemotherapy response [26, 27]. The results of this study showed that VMA was negatively correlated with the efficiency of chemotherapy in patients. In addition, MMP-9 and TIMP-1 also present a similar trend of changes, indicating the great potential of MMP-9 and TIMP-1 as two potential biological indicators for clinical determination of the chemotherapy efficacy. In addition, MMP-9, TIMP-1, and VMA were related to the 2-year survival of patients, which further provided evidence for the monitoring of therapeutic effect on NB patients by using MMP-9 and TIMP-1 [8, 9, 14].

There are still some deficiencies in this study. The number of patients included in this study is small, and the results obtained still require more evidence. In addition, the follow-up time of this study is relatively short, which will be extended to 5-year survival observation in future studies.

In conclusion, surgery plus chemotherapy for children with NB yields a promising clinical efficacy and a favorable surgical resection outcome. MMP-9 and TIMP-1 may be the potential biological indicators for chemotherapy efficiency and have a reference value for following surgical treatment of patients.

## Figures and Tables

**Table 1:** Chemotherapy regimen.

CAV	Cyclophosphamide (CTX) 750–1000 mg/m^2^ i.v. d1 Jiangsu Hengrui Medicine Co., Ltd., H32020857 Vincristine (VCR) 1.5 mg/m^2^ i.v. d1 Guangdong Lingnan Pharmacy Co., Ltd., H20065857 Adriamycin (ADM) 50 mg/m^2^ i.v. d1 Yabao Pharmaceutical Co., Ltd., H20010554	Courses 1, 3, and 5

EP	Etoposide (VP16) 60–80 mg/m^2^ i.v. d1–d5 Qilu Pharmaceutical Co., Ltd., H37023182 Cisplatin (DDP) 20 mg/m^2^ i.v. d1–d5 Yunnan Phytopharmaceutical Co., Ltd., H53021740	Courses 2, 4, and 5

TOPO	Topology (TOPO) 1.2 mg/m^2^ i.v. d1–d5 Jiangsu Hengrui Medicine Co., Ltd., H20000438	Courses 1 and 2

CTX	CTX 250 mg/m^2^ i.v.gtt d1–d5	Courses 1 and 2

CiE	DDP 50 mg/m^2^ i.v.gtt d1–d4 VP16 200 mg/m^2^ i.v.gtt d1–d3	Courses 3 and 5

CPV	CTX 2.1 g/m^2^ i.v. gtt 48 h Pirarubicin 25 g/m^2^ i.v. gtt 48 h Shenzhen Main Luck Pharmaceutical Co., Ltd., H10930106 VCR 0.67 mg/m^2^ i.v.gtt 72 h	Courses 4 and 6

CV	CTX 5 mg/kg d1–d5 VCR 0.05 mg/kg d1	

**Table 2:** General data (mean ± SD (*n*, %)).

General data		Group A (*n* = 50)	Group B (*n* = 42)	*χ* ^2^/*t*	*P*
Gender				0.670	0.413
Male	61 (66.30)	35 (70.00)	26 (61.90)		
Female	31 (33.70)	15 (30.00)	16 (38.10)		

Age (years)	6.6 ± 1.9	7.2 ± 1.9	7.0 ± 2.3	0.457	0.649

INSS classification				1.271	0.260
III	38 (41.30)	18 (36.00)	20 (47.62)		
IV	54 (58.70)	32 (64.00)	22 (52.38)		

Primary site				2.125	0.713
Abdomen	24 (26.09)	10 (20.00)	14 (33.33)		
Mediastinum	15 (16.30)	9 (18.00)	6 (14.29)		
Paranephros	41 (44.57)	24 (48.00)	17 (40.48)		
Spine	7 (7.61)	4 (8.00)	3 (7.14)		
Others	5 (5.43)	3 (6.00)	2 (4.76)		

Metastasis site				2.125	0.713
Bone	49 (53.26)	23 (46.00)	26 (61.90)		
Lymph node	45 (48.91)	25 (50.00)	20 (47.62)		
Liver	24 (26.09)	12 (24.00)	12 (28.57)		
Intracranial	15 (16.30)	6 (12.00)	9 (21.43)		
Others	16 (17.39)	7 (14.00)	9 (21.43)		

INRG				Fisher	0.498
*L*1	2 (2.17)	2 (4.00)	0 (0.00)		
*L*2	90 (97.83)	48 (96.00)	42 (100.00)		

MYCN status				0.497	0.482
Amplified	71 (77.17)	40 (80.00)	31 (73.81)		
Not amplified	21 (22.83)	10 (20.00)	11 (26.19)		

2-year recurrence rate				0.885	0.347
Recurrence	18 (19.57)	8 (16.00)	10 (23.81)		
Nonrecurrence	74 (80.43)	42 (84.00)	32 (76.19)		

2-year prognosis				0.379	0.538
Survival	56 (60.87)	29 (58.00)	27 (64.29)		
Death	36 (39.13)	21 (42.00)	15 (35.71)		

INRG, the International Neuroblastoma Risk Group.

**Table 3:** Chemotherapy effects (*n*, %).

	Group A (*n* = 50)	Group B (*n* = 42)	*χ* ^2^	*P*
CR	33 (66.00)	33 (78.57)	1.779	0.182
PR	9 (18.00)	7 (16.67)	0.028	0.867
SD	2 (4.00)	0 (0.00)	Fisher	0.498
PD	6 (12.00)	2 (4.76)	0.733	0.392
Total effective rate	42 (84.00)	40 (95.24)	1.929	0.165

CR, complete response; PR, partial response; SD, stable disease; PD, progressive disease.

**Table 4:** Surgical resection.

			Group A (*n* = 50)	Group B (*n* = 42)	*χ* ^2^	*P*
After initial chemotherapy first surgery	Complete resection	43 (46.74)	25 (50.00)	18 (42.86)	0.468	0.494
	Majority excision	7 (7.61)	2 (4.00)	5 (11.90)	1.060	0.303
	Incomplete resection	6 (6.52)	3 (6.00)	3 (7.14)	0.041	0.839
	Delayed surgery	36 (39.13)	20 (40.00)	16 (38.10)	0.035	0.852
	Surgical resection range	56 (60.87)	30 (60.00)	26 (61.90)		

Final surgery	Complete resection	16 (17.39)	8 (40.00)	8 (19.05)	0.360	0.549
	Majority excision	11 (11.96)	8 (40.00)	3 (7.14)	1.023	0.312
	Incomplete resection	9 (9.78)	4 (20.00)	5 (11.90)	0.150	0.699
	Delayed surgery	0 (0.00)	0 (0.00)	0 (0.00)		
	Surgical resection range	100 (100.00)	100 (100.00)	100 (100.00)		

Complete resection refers to the resection rate of the lesion exceeding 90%. Majority resection refers to the resection rate of the lesion ranging from 90% to 95%. Incomplete resection refers to the resection rate of the lesion ranging from 50% to 90%. Delayed surgery refers to the expected surgical resection of the lesion range not exceeding 50%.

**Table 5:** Analysis of complication (mean ± SD (*n*, %)).

		Group A (*n* = 50)	Group B (*n* = 42)	*χ* ^2^/*t*	*P*
	Myelosuppression stages I-II	27 (54.00)	21 (50.00)	0.146	0.702

	Myelosuppression stages III-IV	23 (46.00)	21 (50.00)		

CD4+/CD8+	Before chemotherapy	1.71 ± 0.36	1.68 ± 0.39	0.383	0.702
	After chemotherapy	0.81 ± 0.21^*∗*^	0.88 ± 0.21^*∗*^	1.593	0.115
	After surgery	0.41 ± 0.13^*∗*^^#^	0.39 ± 0.21^*∗*^^#^	0.558	0.578

NK cells	Before chemotherapy	8.86 ± 1.14	8.72 ± 0.90	0.645	0.521
	After chemotherapy	12.10 ± 1.96^*∗*^	12.21 ± 2.28^*∗*^	0.249	0.804
	After surgery	21.79 ± 5.13^*∗*^^#^	23.15 ± 4.84^*∗*^^#^	1.300	0.197

^*∗*^Comparison with the same group before chemotherapy, *P* < 0.05. ^#^Comparison with the same group after chemotherapy, *P* < 0.05.

## Data Availability

The datasets used during the present study are available from the corresponding author upon request.
